# Circulating Fibroblast Activation Protein as Potential Biomarker in Patients With Inflammatory Bowel Disease

**DOI:** 10.3389/fmed.2021.725726

**Published:** 2021-09-21

**Authors:** Fabio Corsi, Luca Sorrentino, Sara Albasini, Francesco Colombo, Maria Cigognini, Alessandro Massari, Carlo Morasso, Serena Mazzucchelli, Francesca Piccotti, Sandro Ardizzone, Gianluca M. Sampietro, Marta Truffi

**Affiliations:** ^1^Breast Unit, Surgery Department, Istituti Clinici Scientifici Maugeri IRCCS, Pavia, Italy; ^2^Department of Biomedical and Clinical Sciences “L. Sacco”, Universitá di Milano, Milan, Italy; ^3^Division of General Surgery, ASST Fatebenefratelli Sacco, Luigi Sacco University Hospital, Milan, Italy; ^4^Division of General Surgery, ASST Rhodense, Rho Memorial Hospital, Milan, Italy; ^5^Division of Gastroenterology, ASST Fatebenefratelli Sacco, Luigi Sacco University Hospital, Milan, Italy; ^6^Nanomedicine and Molecular Imaging Lab, Istituti Clinici Scientifici Maugeri IRCCS, Pavia, Italy

**Keywords:** inflammatory bowel disease, fibroblast activation protein, blood biomarkers, diagnosis, mucosal healing, chronic patient

## Abstract

A major concern in the management of Inflammatory Bowel Disease (IBD) is the absence of accurate and specific biomarkers to drive diagnosis and monitor disease status timely and non-invasively. Fibroblast activation protein (FAP) represents a hallmark of IBD bowel strictures, being overexpressed in stenotic intestinal myofibroblasts. The present study aimed at evaluating the potential of circulating FAP (cFAP) as an accessible blood biomarker of IBD. Quantitative determination of cFAP was performed by enzyme-linked immunosorbent assay on plasma samples prospectively collected from patients with IBD and control subjects. A discrimination model was established on a training set of 50% patients and validated on independent samples. Results showed that cFAP concentration was reduced in patients with IBD when compared to controls (*p* < 0.0001). Age, sex, smoking, disease location and behavior, disease duration and therapy were not associated with cFAP. The sensitivity and specificity of cFAP in discriminating IBD from controls were 70 and 84%, respectively, based on the optimal cutoff (57.6 ng mL^−1^, AUC = 0.78). Predictions on the test set had 57% sensitivity, 65% specificity, and 61% accuracy. There was no strong correlation between cFAP and routine inflammatory markers in the patients' population. A subgroup analysis was performed on patients with Crohn's disease undergoing surgery and revealed that cFAP correlates with endoscopic mucosal healing. In conclusion, cFAP deserves attention as a promising blood biomarker to triage patients with suspected IBD. Moreover, it might function as a biomarker of post-operative remission in patients with Crohn's disease.

## Introduction

Currently, no accurate serum biomarker of inflammatory bowel disease (IBD) is available to aid clinicians in establish a diagnosis properly (1–3). As a consequence, several patients complaining about gastrointestinal symptoms undergo invasive and costly diagnostic procedures, and only a small subset of them receives a diagnosis of IBD (4, 5). Definitive diagnosis relies on the histological assessment of bowel biopsies from endoscopy, which remains highly uncomfortable for patients and requires expert gastroenterologists and pathologists. In addition, a diagnostic delay frequently triggers a delay in the establishment of appropriate therapies, with an impact on disease progression and increased risk for complications.

A few serum biomarkers have been described in the literature, although controversial results about their utility exist (2, 6, 7). Among them, the anti-*Saccharomyces cerevisiae* antibody (ASCA) is the most well-known. However, its sensitivity is not optimal (~39–44%). Perinuclear antineutrophil cytoplasmic antibodies (pANCAs) are frequently found in serum samples from patients with ulcerative colitis (UC), but they are less frequent in patients with Crohn's disease (CD) (8, 9). Therefore, ASCA and pANCAs are used in combination to better define the IBD type affecting the patient, rather than to diagnose IBD itself. Patients with ASCA+/pANCA- are more likely to have CD, but the sensitivity achieved by the combined use of these two markers is 55%. Indeed, a generally low sensitivity limits the overall utility of the identified serological markers for IBD diagnosis.

Fecal markers, such as fecal calprotectin (FC), appear more specific for intestinal inflammation. FC dosage may be helpful in the evaluation of disease exacerbations and monitoring of therapy responsiveness. However, FC cannot distinguish IBD from other causes of intestinal inflammation, and it is strongly associated with colonic inflammation, though much less with ileal inflammation (6, 10, 11). Furthermore, considerable intra-individual variability of FC levels is observed, thus adding critical issues for the correct interpretation of the results (12, 13).

Repeated endoscopy is required not only for IBD diagnosis or surveillance, but it also allows to follow up medically or surgically treated IBD patients, especially patients with CD undergoing surgery. Recently, mucosal healing (MH) has been suggested as the real therapeutic goal in these patients, as it is associated with less frequent relapses, reduced hospitalization and lower risk of further surgery (14). However, the only way to assess MH is currently ileocolonoscopy, and both clinicians and patients would highly desire a less invasive biomarker.

In the last years, fibroblast activation protein (FAP) has been identified as a hallmark of intestinal fibrosis in CD (15–17). FAP is an inducible cell surface glycoprotein belonging to the postprolyl dipeptidyl aminopeptidase enzyme family, and it is a well-recognized marker of reactive fibroblasts in different contexts (18). In CD, FAP expression is specifically up-regulated on intestinal strictured myofibroblasts (15, 16, 19). FAP also exists as a soluble enzymatically active form, which can be detected in human blood. Some studies have associated altered circulating FAP (cFAP) levels with certain disorders, such as cancer and liver fibrosis (20, 21). However, the significance of cFAP in IBD has never been explored.

The present study aimed to investigate the potential of cFAP as a reliable serological biomarker of IBD, assessing its plasma levels in a cohort of patients with IBD vs. control subjects. Moreover, a subgroup analysis was performed on a subset of patients with CD undergoing surgery to correlate cFAP with post-operative endoscopic disease activity.

## Materials and Methods

### Patient Population

From April 2018 to February 2020, all consecutive patients affected by IBD and referred to the ASST Fatebenefratelli Sacco, “Luigi Sacco” University Hospital (Milano, Italy) were eligible to participate in the study. Inclusion criteria were: proven histopathological diagnosis of CD or UC, any disease pattern and localization, 18–85 years old. Patients were excluded from the study if they had an unclear IBD diagnosis (indeterminate colitis), displayed rheumatologic disease, chronic liver diseases, chronic heart failure or other concurrent gastrointestinal and autoimmune diseases. Two cohorts of patients were considered: (1) patients with controlled IBD undergoing routine outpatient evaluation; (2) patients with active IBD undergoing surgery for complicated disease. Indication for surgery was established during a formal multidisciplinary meeting involving gastroenterologists, surgeons, pathologists, and radiologists. Patients were excluded if they were referred in emergency, if they displayed severe sepsis, and in case they were under steroids in the last month or under immunosuppressants or anti-TNF antibodies in the last 3 months. A control group was formed of healthy volunteers without any gastrointestinal or autoimmune disorder. In addition, a cohort of patients with diverticulitis was enrolled as part of the study to make a comparison between IBD and another intestinal disease (baselines features of patients with diverticulitis are reported in Supplementary Table 1).

### Blood Samples Collection and cFAP Detection

From each subject, 10 mL blood sample was collected in EDTA-coated tubes at the time of outpatient visit or as part of the pre-operative assessment in case patients underwent surgery. Plasma was isolated by centrifuge at 1,000 × g for 10 min, transferred in sterile vials and stored at −80°C until usage. FAP concentration was determined by double-antibody sandwich enzyme-linked immunosorbent assay, according to the manufacturer's instructions (Human FAP DuoSet ELISA, R&D systems). Each plasma sample was diluted in Reagent Diluent (1:200) and run in a 96-well microplate as duplicates. A calibration curve was performed using seven-point dilutions of recombinant human FAP as standard. Absorbance was read using a testing wavelength of 450 nm and a correction wavelength of 570 nm. The intra-assay coefficient of variability (CV) was 2.8% (±0.6, *n* = 14); the inter-assay CV was 5.4% (±2.4, *n* = 10).

### Clinical Assessment

For all the patients, demographic and clinical data were collected at baseline in a prospective database. Age at diagnosis, disease location and clinical phenotypes were evaluated with the Montreal classification (22). Laboratory data on blood analyses were exported as electronic medical record from the hospital management system (clinical electronic repositories). For a subgroup of patients with CD undergoing ileocolonic resection, endoscopic procedures were performed at 12 months after surgical intervention, in the setting of routine clinical practice. The endoscopic reports were reviewed by an IBD physician to grade endoscopic activity through Rutgeerts score. Scores of i0 and i1 were regarded as post-surgery remission; scores of i2, i3, and i4 were considered post-surgery recurrence. At the time of endoscopy, a second blood sample was withdrawn from the patient and analyzed for cFAP as previously described. Rutgeerts score and paired cFAP dosage were analyzed by Spearman's rank correlation coefficient.

### Statistical Methods

Variables were reported as means ± standard deviations (SD) or as absolute numbers and percentages. Categorical variables were compared using χ^2^-test or exact Fisher's, while continuous variables were compared using Student's *T*-test or non-parametric Wilcoxon test in case of non-normal distribution of the variable. If it was necessary to apply regression models on non-normal variables, an appropriate transformation was applied to make them follow a Gaussian distribution.

To define a diagnostic model, the original dataset was divided into two independent samples with the same size. To this aim, temporal criterion was used as previously described: (23) the first half of enrolled patients formed the training set; the second half of enrolled patients generated the test set. The first sample was used to develop the diagnostic model. In order to estimate the diagnostic accuracy, the area under curve (AUC) of the receiver operating characteristic (ROC) curve was designed. An internal validation for AUC was performed with bootstrap method. Briefly, the original patient population was re-sampled 500 times and the optimism index (the mean of differences between AUC on bootstrap sample and AUC on original sample) was calculated. Optimism is the amount by which the AUC (or “the apparent prediction accuracy”) overestimates the true prediction accuracy of the model. Then, the corrected AUC after bootstrap was reported. The second sample was used to externally validate the developed diagnostic model in order to evaluate its performance (accuracy, sensitivity, specificity, positive/negative predictive value) in other independent dataset and to determine generalizability of the derived diagnostic rule to new patients.

Data analysis was performed using SAS software (v. 9.4, SAS Institute Inc., Cary, USA) and R software (v. 3.5.1, © The R Foundation).

### Ethics Approval

The study was authorized by the Ethical Committee of ASST Fatebenefratelli Sacco (Milano, Area 1) as protocols n. 545/2016 and n. 24916/2019. The study protocol was conducted in accordance with the International Conference on Harmonization (ICH) Good Clinical Practice (GCP) guidelines. Informed consent was obtained from each subject included in the study.

## Results

### Baseline Characteristics of Study Population

A total of 432 subjects were included in the study: 272 patients had IBD, and 160 were healthy controls (HC). Patients with IBD attending routine outpatient consultation (*n* = 152) and patients with IBD undergoing surgery (*n* = 120) were analyzed separately not to introduce any bias due to the non-homogeneity of disease status among the two groups. In the first group, 86 patients (56.6%) had CD and 66 patients (43.4%) had UC. Their mean age was 46.8 (±14.7) years, 60 patients (39.5%) were female, 92 (60.5%) were male, 64 patients (42.1%) were smokers. The mean disease duration was 11.6 (±8.0) years. In the second group, 96 patients (80.0%) had confirmed diagnosis of CD and 24 (20.0%) of UC. Their mean age was 46.2 (±16.1) years; 47 patients (39.2%) were female, and 73 (60.8%) were male. Thirty-six patients (30%) declared to be smokers. The mean disease duration was 12.0 (±10.1) years. The HC group had an average age of 44.2 (±16.0) years and displayed a slight prevalence of females (62.5%) than males (37.5%). In the group, 54 people (33.8%) were smokers. HC and IBD groups were similar in terms of age (*p* = 0.22) and smoking habitude (*p* = 0.21). There was a male predominance in the IBD groups as compared with HC (*p* < 0.0001), while no different gender distribution was observed in the two IBD groups (*p* = 0.96). The distribution of baseline variables in the study population is shown in Table 1.

**Table 1:** Baseline variables of the two cohorts of patients with IBD and of the healthy controls (HC) included in the study.

**Variable**	**HC**	**IBD no surgery**	**IBD surgery**	***p*-value**
	**(*n* = 160)**	**(*n* = 152)**	**(*n* = 120)**	
**Age** **(mean** **±** **SD, years)**	44.2 ± 16.0	46.8 ± 14.7	46.2 ± 16.1	0.22
**Gender**, ***n*****(%)**				
Female	100 (62.5)	60 (39.5.)	47 (39.2)	<0.0001
Male	60 (37.5)	92 (60.5)	73 (60.8)	
**Smoke**, ***n*****(%)**				
Yes	62 (38.8)	64 (42.1)	38 (31.7)	0.21
No	98 (61.2)	88 (57.9)	82 (68.3)	

*Age of patients is expressed as continuous variable (mean ± SD); gender and smoke habit are expressed as total number of subjects and frequency distribution*.

### Plasma FAP in Patients With IBD

**Figure 1:**
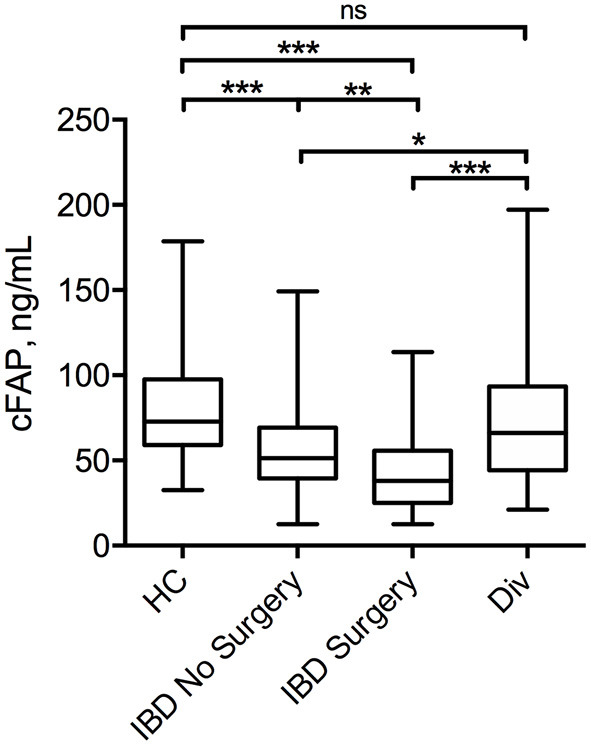
Box plot displaying the concentration of cFAP in patients with IBD attending routine outpatient consultation (*n* = 152) or undergoing surgery (*n* = 120) as compared with healthy controls (HC, *n* = 160) and patients with diverticulitis (Div, *n* = 20). Statistical analysis was performed by Student *t*-test, *p = 0.04; **p < 0.001; ***p < 0.0001.

Mean cFAP concentration was significantly lower in patients with IBD, both in the group attending outpatient visit (55.7 ± 26.8 ng mL^−1^) and in the surgery group (42.4 ± 26.7 ng mL^−1^) than in HC (76.5 ± 32.5 ng mL^−1^, *p* < 0.0001) (Figure 1). Levels of cFAP in patients with IBD undergoing surgery were reduced as compared to patients with stable disease that did not require surgery (*p* < 0.001). There was no correlation between cFAP levels and age across the groups (ρ = 0.02, *p* = 0.78 in HC; ρ = 0.11, *p* = 0.18 in patients with IBD attending outpatient visit; ρ = 0.08, *p* = 0.39 in patients with IBD undergoing surgery). Levels of cFAP were not significantly associated with sex (*p* = 0.09, *p* = 0.19, and *p* = 0.58 in HC and IBD groups, respectively). Moreover, age at diagnosis, location and behavior of the disease, disease duration or biological therapy were not related to cFAP, thus not ascribing cFAP reduction to some specific clinical features but rather to the presence of IBD (Table 2). Patients with CD and patients with UC were both characterized by reduced levels of cFAP as compared to controls (*p* < 0.0001). No significant cFAP difference was observed between CD and UC subtypes in the group of IBD attending outpatient visit (52.9 ± 22.2 ng mL^−1^ in CD, 59.3 ± 31.7 ng mL^−1^ in UC, *p* = 0.14) and in IBD undergoing surgery (42.2 ± 24.7 in CD, 43.4 ± 33.8 in UC, *p* = 0.83, Supplementary Figure 1). Disease features from patients with CD and UC were analyzed separately and showed no dependence on cFAP levels (see Supplementary Table 2).

**Table 2:** Baseline characteristics and levels of cFAP in HC and IBD groups.

**Variable**	**HC (** ***n*** **=** **160)**			**IBD no surgery (** ***n*** **=** **152)**			**IBD surgery (** ***n*** **=** **120)**		
	***n* (%)**	**cFAP (ng mL^**−1**^)**	***p*-value**	***n* (%)**	**cFAP (ng mL^**−1**^)**	***p*-value**	***n* (%)**	**cFAP (ng mL^**−1**^)**	***p*-value**
**Gender**									
Female	100 (62.5)	72.6 ± 30.1	0.09	60 (39.5)	52.4 ± 24.6	0.23	47 (39.2)	39.6 ± 17.7	0.58
Male	60 (37.5)	83.1 ± 32.2		92 (60.5)	57.8 ± 28.1		73 (60.8)	43.7 ± 23.8	
**Smoking**									
Yes	62 (38.8)	74.9 ± 37.1	0.28	64 (42.1)	55.5 ± 26.8	0.96	38 (31.7)	38.7 ± 23.7	0.07
No	98 (61.2)	77.6 ± 29.4		88 (57.9)	55.8 ± 27.0		82 (68.3)	43.6 ± 20.6	
**Family history of IBD**									
Yes				17 (12.2)	56.9 ± 21.9	0.94	16 (13.8)	35.9 ± 16.5	0.25
No				122 (87.8)	56.4 ± 27.1		100 (86.2)	42.8 ± 21.3	
**Disease duration**									
≤ 10 years				73 (51.8)	56.5 ± 29.2	0.42	56 (50.5)	42.0 ± 21.4	0.96
>10 years				68 (48.2)	53.0 ± 22.2		55 (49.5)	41.2 ± 18.7	
**Age at diagnosis**									
A1, <16 years				7 (4.9)	51.0 ± 18.5	0.64	14 (12.1)	41.9 ± 20.1	0.44
A2, 17–40 years				93 (64.6)	53.8 ± 26.3		66 (56.9)	40.3 ± 21.4	
A3, >40 years				44 (30.5)	57.8 ± 26.1		36 (31)	44.6 ± 20.3	
Montreal location^*^									
L1, terminal ileum				23 (27.4)	51.6 ± 22.2	0.23	62 (66)	41.0 ± 17.9	0.44
L2, colon				10 (11.9)	66.9 ± 31.6		9 (9.6)	51.3 ± 29.5	
L3, ielocolon				43 (51.2)	51.9 ± 20.6		21 (22.3)	40.3 ± 22.1	
L4, upper gastrointestinal tract				8 (9.5)	50.0 ± 10.3		2 (2.1)	55.6 ± 10.4	
Montreal behavior^*^									
B1, non-stricturing non-penetrating				36 (43.4)	52.5 ± 23.5	0.71	5 (5.3)	58.4 ± 37.4	0.67
B2, stricturing				35 (42.2)	56.0 ± 20.9		43 (45.7)	41.4 ± 17.8	
B3, penetrating				12 (14.4)	50.8 ± 21.5		46 (49)	41.1 ± 19.6	
Perianal disease^*^									
Yes				8 (10.3)	34.2 ± 23.5	0.006	21 (20)	36.1 ± 15.2	0.14
No				70 (89.7)	61.8 ± 26.8		68 (80)	43.6 ± 19.9	
**Montreal extent^#^**									
E1, proctitis				7 (12.5)	39.4 ± 16.4	0.16	2 (9.5)	45.5 ± 7.9	0.22
E2, left-sided colitis				19 (29.7)	64.5 ± 37.1		1 (4.8)	70.8 (± 0.0)	
E3, extensive colitis				37 (57.8)	59.5 ± 30.2		18 (85.7)	34.8 ± 19.0	
**Medications**									
Biological therapy				119 (88.8)	55.6 ± 24.8	0.71	32 (27.1)	39.3 ± 20.5	0.01
Other therapy				9 (6.7)	51.1 ± 27.7		50 (42.4)	37.7 ± 20.1	
None				6 (4.5)	62.1 ± 35.7		36 (30.5)	49.3 ± 19.9	

*cFap is expressed as mean ± SD*.

* *Applicable to patients with CD only*.

# *Applicable to patients with UC only*.

In order to investigate whether cFAP is specific for IBD or a marker common with other intestinal diseases, we measured cFAP levels also in a cohort of patients with diverticulitis (*n* = 20). The results, reported in Figure 1, showed that cFAP concentration in diverticulitis is not significantly different from that measured in HC (*p* = 0.27). By contrast, it is significantly higher than cFAP levels observed in IBD (*p* = 0.04 vs. IBD with controlled disease; *p* < 0.0001 vs. IBD undergoing surgery).

### Diagnostic Value of cFAP

**Figure 2:**
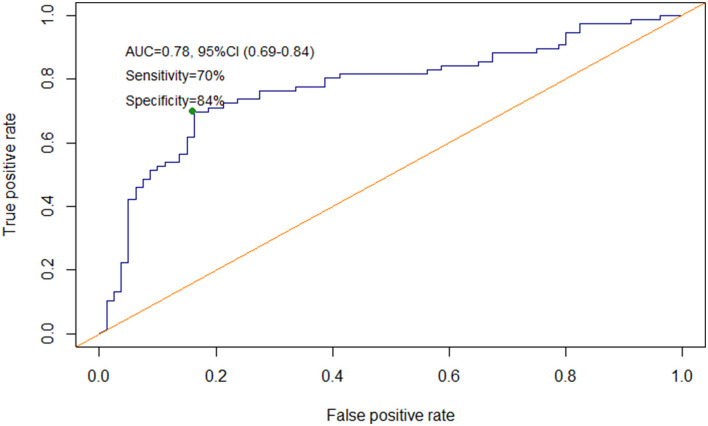
Receiver operating characteristic (ROC) curve for cFAP level's accuracy to distinguish patients with IBD from HC.

To investigate whether cFAP could help to discriminate patients with IBD from HC, a diagnostic model was set up. In order to avoid any bias due to complicated disease, patients with IBD undergoing surgery were excluded from this analysis. A ROC curve was computed using data from HC and patients with IBD assigned to the training set (*n* = 156). The analysis showed that cFAP is able to distinguish patients with IBD from HC with an AUC of 0.78 (CI 0.69–0.84). The sensitivity of cFAP was as high as 70%, and the specificity reached 84% based on the optimal cut-off (57.6). cFAP was able to identify true IBD cases with a positive predictive value (PPV) of 80.3% and a negative predictive value (NPV) of 74.4%. The optimism index was equal to 0.01; the calculated AUC after bootstrap was 0.77 (Figure 2). The as-designed diagnostic model was applied to an independent set of patients (*n* = 156), used for validation. The discrimination matrix revealed a capability to differentiate IBD and HC with 57% sensitivity, 65% specificity, and 61% accuracy (Supplementary Figure 2).

### Association Between cFAP and Other Inflammatory Markers

Association studies were performed between cFAP levels and the main routine markers of inflammation from blood analysis in the patients' population. The analysis showed a weak inverse correlation between cFAP and erythrocyte sedimentation rate {ESR, *r* = −0.31 [C.I. (−0.47, −0.13), *p* = 0.0008, *n* = 116]}, and between cFAP and C-reactive protein {CRP, *r* = −0.39 [C.I. (−0.51, −0.27), *p* < 0.0001, *n* = 211]}. No other significant correlations were observed with total white blood cells, the relative amount of neutrophils, nor markers derived from protein electrophoresis in peripheral blood (see Supplementary Table 3). Beyond blood markers, the dosage of FC was evaluated as an additional measure of intestinal inflammation activity. FC did not show any correlation with cFAP values in the study population {*r* = −0.14 [C.I. (−0.39, 0.13), *p* = 0.29, *n* = 59]}.

### cFAP as Biomarker of Recurrence in CD

**Figure 3:**
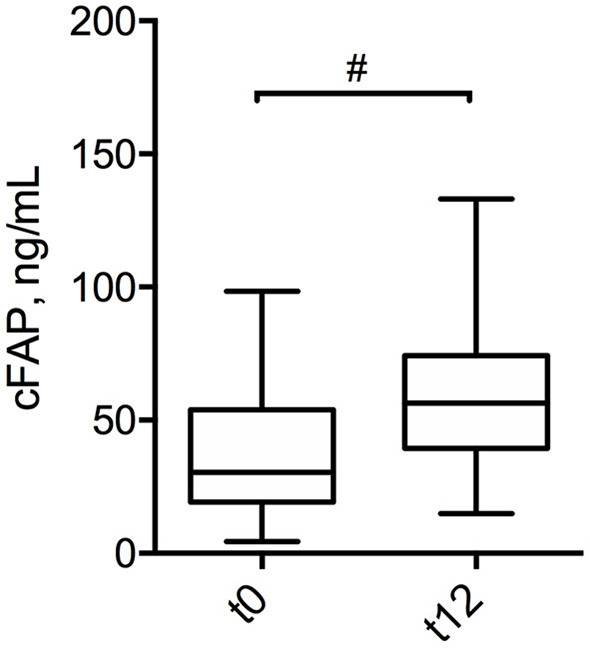
Box plot displaying the concentration of cFAP in patients with CD at pre-operative stage (t0) and at 12 months post-surgery (t12). Statistical analysis was performed by paired Wilcoxon test, ^#^*p* = 0.02.

**Figure 4:**
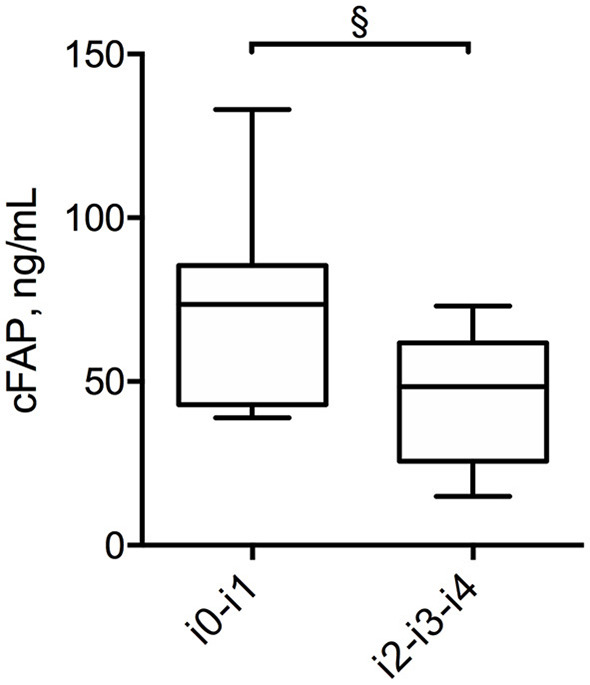
Box plot displaying the concentration of cFAP in patients with CD who have attained endoscopic remission (Rutgeerts score i0, i1) and in those with post-surgery recurrence (Rutgeerts score i2, i3, i4). Statistical analysis was performed by Mann Whitney test, ^§^*p* = 0.03.

In order to further analyze the potential of cFAP as a biomarker of IBD, a subgroup analysis was performed in 21 patients with CD who concluded a regular follow up of at least 12 months post-surgery. Baseline characteristics of this subgroup of patients with CD is shown in Supplementary Table 4. Mean cFAP concentration was significantly increased at 12 months post-surgery as compared to preoperative values (*p* = 0.02, Figure 3). Interestingly, there was a significant inverse correlation between cFAP dosage and disease activity at 12 months post-surgery, as graded by Rutgeerts score upon endoscopic examination {*r* = −0.52 [C.I. (−0.78, −0.09), *p* = 0.017]}. Higher cFAP values were associated with lower scores, thus suggesting that increased cFAP could be a biomarker of post-operative remission. We thus compared cFAP concentrations in those patients who attained endoscopic remission (Rutgeerts i0, i1) vs. those who experienced recurrence at 12 months post-surgery (Rutgeerts i2, i3, i4). Data showed that cFAP was significantly increased in patients with endoscopically-assessed remission (*p* = 0.03, Figure 4).

## Discussion

Early and definite diagnosis of IBD is a major point of concern for clinical management of these disorders (4, 24–26). Currently, IBD diagnosis is based on the complex interpretation of history, clinical signs, endoscopic and histopathologic data from biopsies, whose reliability often depends on extremely few expert operators on the territory. Therefore, easy-accessible blood biomarkers would be a tool of paramount importance for clinicians to timely triage patients suspected to have IBD, before prescribing more invasive and costly imaging procedures in a reference center (1–3).

In this study, the plasma levels of FAP were analyzed in patients with IBD and found to be significantly reduced compared to HC (Figure 1). Reduced cFAP levels were confirmed in both patients with CD and UC. Moreover, no correlations between cFAP and any recorded disease characteristic were found, thus supporting the hypothesis that cFAP is a general marker of IBD rather than an indicator of a particular IBD subtype or pattern. In the present study, we have included patients with controlled IBD under treatment and patients with active IBD undergoing surgery. Surgery in IBD is indicated when an aggressive disease presents with symptoms, or in case of uncontrolled disease after therapy failure, as often happens with UC or stricturing CD. Our findings revealed that cFAP is lower in both IBD cohorts as compared to HC. Furthermore, cFAP was significantly lower in patients undergoing surgery than patients with controlled disease (*p* < 0.0001), thus suggesting a role for cFAP as a biomarker of IBD activity and remission: the more cFAP is reduced, the more disease is active. By contrast, patients with diverticulitis had similar cFAP levels than HC. Despite preliminary, this observation indicates that reduced cFAP may be a specific marker of IBD over other inflammatory gastrointestinal disorders.

Other observations in the literature already reported reduced levels of cFAP in pathological contexts characterized by stroma reactivity, such as some cancers and myocardial infarction (27). To the best of our knowledge, this is the first investigation of cFAP in a cohort of patients with IBD. The cause for reduced cFAP level still remains poorly elucidated, but it could be that a systemic reaction to the disease occurs. Indeed, the origin of cFAP is not completely clarified (28, 29). FAP extracellular domain can be shed from cells as soluble form, but cause and actors involved in this proteolytic cleavage have not been demonstrated yet. Activated fibroblasts, myofibroblasts, and the hepatobiliary system appear as the primary physiological sources of FAP, though it is likely that multiple organs may contribute to the circulating levels of FAP (27, 30). There is literature showing that proteins of the dipeptidyl peptidase family, such as the FAP paralog DPP4, are expressed in gut epithelial cells and that their expression increases in cells that display an enterocytic differentiation (31–33). Since enterocytes are damaged and partly destroyed in IBD, it could be hypothesized that one of the cellular sources of these proteins fails to produce and secrete the proteins to the same extent as it happens in the healthy physiological state. However, studies on FAP secretion by enterocytes in IBD are lacking and this hypothesis cannot yet be confirmed. Moreover, results from our study revealed that patients with stricturing disease had similar cFAP levels compared to penetrating disease and non-stricturing non-penetrating disease, both in IBD patients controlled with therapy (*p* = 0.71) and in patients treated by surgery (*p* = 0.67). This finding is particularly relevant, since it further suggests that cFAP could be not only related with the fibroblasts' local presence in bowel lesions, but also with other unknown pathways related to FAP independent from strictures. Further preclinical studies are required to uncover the mechanisms responsible for cFAP production and to explain why it is reduced in IBD.

In the present study, a predictive diagnostic model based on ROC curve assessed the capability of cFAP to discriminate IBD and healthy groups with 78.0% accuracy (Figure 2). In our tested population, cFAP was able to identify real IBD cases with a PPV of 80.3% and a NPV of 74.4%. Despite preliminary, these results suggest that cFAP could be a valuable, non-invasive solution to triage patients suspected to have IBD in primary care diagnostic. The future clinical potential of cFAP may be intended to accelerate clinical diagnosis for patients ending up with reduced cFAP, who will promptly undergo more invasive and costly imaging procedures. The absence of any strong correlation between cFAP and traditional though a specific inflammatory markers, such as ESR, CRP, FC, indicates that cFAP may be a more specific IBD biomarker than other aspecific inflammatory indexes. It also means that information derived from cFAP is different and non-redundant with currently available inflammatory markers.

It has to be noted that discriminative performances deriving from predictions on the independent test set achieved 57% sensitivity, 65% specificity, and 61% accuracy. These parameters certainly highlighted some limitations of cFAP as a stand-alone biomarker for IBD diagnosis. However, a performance's decrease is often expected in external validation and accuracy remains significantly higher than the null model (AUC = 61% with CI 0.54–0.72). Moreover, we need to consider that no other single serological test is currently available to guide IBD diagnosis in primary care. The Prometheus IBD Sgi diagnostic^®^ combines serologic, genetic and inflammatory markers to aid decision-making in IBD diagnosis. Despite being welcomed as a “holy Graal,” this multi-marker panel presents several concerns. First, only three markers appeared as really predictive of IBD: pANCA, ASCA IgA, and ASCA IgG (34). Secondly, the accuracy of its serologic markers was assessed in cohorts with a high prevalence of IBD (up to 62%), thus its value in a real-world setting with a low-prevalence of IBD remains controversial (35, 36). Recently, the Prometheus test was applied in a series of patients with IBD seen at a tertiary referral center. The sensitivity for CD was 52%, with an accuracy of 61.5%. A better performance was observed for UC (sensitivity 67%, accuracy 80%), but the overall conclusion was that the test is not robust enough for initial diagnostics of IBD (37). In this context, cFAP could be extremely promising, mainly because it represents a much simpler dosage of a single plasma protein, which has high relevance for IBD pathophysiology. After the present study, further trials, including other centers and community hospitals, should be conducted to validate FAP as a blood biomarker of IBD.

Interestingly, in the present study, increased cFAP was demonstrated to be associated to MH in patients with CD treated by surgery and undergoing follow-up ileocolonoscopy (*p* = 0.03, Figure 4). Patients with UC requiring surgery were excluded from this analysis because, once operated of proctocolectomy, these patients should be considered cured, so any recurrence is not expected. MH is currently considered the therapeutic goal for patients with CD, and today it is the endpoint of several trials to estimate the success rate of novel therapies (38, 39). However, the definition of MH is quite ambiguous, depending on precise endoscopic evaluation and reporting. Recently, a combined blood test called the endoscopic healing index has been developed to assess endoscopic remission, but it requires the quantitative determination of 13 different proteins (40). Preliminary results from our study suggest that cFAP might deserve attention as an ease-to-get, stand-alone blood biomarker of MH after surgery in CD, since a concordance rate with endoscopic findings was found. A limitation for this observation consists in the small number of patients for which endoscopic data were available at 12 months post-surgery. A larger study with a longer longitudinal follow up is now required to confirm the observed correlation and validate cFAP as a biomarker of post-operative MH.

In conclusion, the present study provides evidence that cFAP is reduced in patients with IBD as compared to controls. Since no accurate serum biomarker of IBD is currently available, cFAP deserves attention as a potential non-invasive solution to triage patients with suspected IBD. Moreover, this study provides a preliminary indication that cFAP increases in patients with CD experiencing endoscopic remission, thus suggesting exploration of this protein as a novel biomarker of MH.

## Data Availability Statement

The original contributions presented in the study are included in the article/Supplementary Material, further inquiries can be directed to the corresponding author.

## Ethics Statement

The studies involving human participants were reviewed and approved by the Ethical Committee of ASST Fatebenefratelli Sacco (Milano, Area 1) as protocols n. 545/2016 and n. 24916/2019. The patients/participants provided their written informed consent to participate in this study.

## Author Contributions

FCor and MT planned the study. FCol, SAr, and GS recruited patients. MC, AM, CM, SM, FP, and MT collected data. FCor, LS, SAl, FCol, and MT analyzed data. FCor, SAl, and MT drafted the manuscript. All the authors have approved the final draft submitted.

## Funding

This study was supported by Università di Milano, Piano di Sostegno della Ricerca (Linea 2 to FCor and SAr). Article processing fee was covered by Istituti Clinici Scientifici Maugeri IRCCS.

## Conflict of Interest

The authors declare that the research was conducted in the absence of any commercial or financial relationships that could be construed as a potential conflict of interest.

## Publisher's Note

All claims expressed in this article are solely those of the authors and do not necessarily represent those of their affiliated organizations, or those of the publisher, the editors and the reviewers. Any product that may be evaluated in this article, or claim that may be made by its manufacturer, is not guaranteed or endorsed by the publisher.
