# Supplementary material for: Circulating Fibroblast Activation Protein as Potential Biomarker in Patients With Inflammatory Bowel Disease
Source: Front Med (Lausanne). 2021 Sep 21;8:725726. doi: 10.3389/fmed.2021.725726 (PMC8490650; doi:10.3389/fmed.2021.725726)
Supplement: Supplementary file 1 [file Data_Sheet_1.docx]

Supplementary Material

# Supplementary Figures and Tables

## Supplementary Tables

| **Variable** | **Diverticulitis (n=20)** |
| --- | --- |
| **Age (mean ± SD, years)** | 60.5 ± 13.1 |
| **Gender, n (%)** |  |
| Female | 6 (30.0) |
| Male | 14 (70.0) |
| **Smoke** |  |
| Yes | 8 (40.0) |
| No | 12 (60.0) |

**Supplementary Table 1.** Distribution of baseline variables in patients with diverticulitis enrolled in the study (n=20). Age of patients is expressed as continuous variable (mean ± SD); all the other variables are shown as total number of subjects and frequency distribution

|  | | | | | | |
| --- | --- | --- | --- | --- | --- | --- |
| **Variable** | **IBD No Surgery (n=152)** | | | | | |
|  | **CD (n=86)** | | | **UC (n=66)** | | |
|  | **n (%)** | **cFAP (ng mL^-1^)** | **p-value** | **n (%)** | **cFAP (ng mL^-1^)** | **p-value** |
| **Gender** |  |  |  |  |  |  |
| Female | 32 (37.2) | 52.8 ± 23.0 | 0.97 | 28 (42.2) | 52.0 ± 26.7 | 0.11 |
| Male | 54 (62.8) | 53.0 ± 23.0 |  | 38 (57.6) | 64.7 ± 34.2 |  |
| **Age** |  |  |  |  |  |  |
| <=45 years | 46 (53.5) | 46.6 ± 18.9 | 0.005 | 22 (33.3) | 55.8 ± 33.9 | 0.53 |
| >45 years | 40 (46.4) | 60.1 ± 23.8 |  | 44 (66.7) | 61.1 ± 30.7 |  |
| **Smoking** |  |  |  |  |  |  |
| Yes | 44 (51.2) | 51.9 ± 22.1 | 0.67 | 25 (37.9) | 60.8 ± 32.3 | 0.76 |
| No | 42 (48.8) | 53.9 ± 22.6 |  | 41 (62.1) | 58.4 ± 31.6 |  |
| **Family history of IBD** |  |  |  |  |  |  |
| Yes | 12 (15.6) | 61.3 ± 21.8 | 0.20 | 57 (91.9) | 60.7 ± 32.3 | 0.33 |
| No | 65 (84.4) | 52.6 ± 21.2 |  | 5 (8.1) | 46.5 ± 20.4 |  |
| **Disease duration** |  |  |  |  |  |  |
| <=10 years | 38 (46.3) | 53.1 ± 21.6 | 0.83 | 35 (59.3) | 60.3 ± 35.6 | 0.22 |
| >10 years | 44 (56.7) | 54.1 ± 23.1 |  | 24 (40.7) | 51.1 ± 20.8 |  |
| **Age at diagnosis** |  |  |  |  |  |  |
| A1 | 7 (8.4) | 51.0 ± 18.5 | 0.18 | 0 (0.0) | - | 0.67 |
| A2 | 60 (72.3) | 51.2 ± 21.7 |  | 33 (54.1) | 58.4 ± 33.1 |  |
| A3 | 16 (19.3) | 62.7 ± 24.4 |  | 28 (45.9) | 55.1 ± 27.1 |  |
| **Medications** |  |  |  |  |  |  |
| Biological therapy | 74 (92.5) | 52.3 ± 20.9 | 0.38 | 45 (83.3) | 61.1 ± 29.6 | 0.50 |
| Other therapy | 2 (2.5) | 67.3 ± 23.2 |  | 7 (13.0) | 46.4 ± 28.7 |  |
| None | 4 (5.0) | 63.7 ± 26.1 |  | 2 (3.7) | 58.9 ± 65.7 |  |

**Supplementary Table 2.** Baseline levels of cFAP in patients with CD and patients with UC included in the cohort of IBD attending outpatient visit. cFap is expressed as mean concentration ± SD

| **Parameter** | **Spearman coefficient (confidential interval)** | **p-value** |
| --- | --- | --- |
| White Blood Cells (n=225) | -0.22 (C.I. -0.34, 0.08) | 0.001 |
| % Neutrophils (n=149) | -0.31 (C.I. -0.45, 0.15) | 0.0001 |
| % Lymphocytes (n=149) | 0.31 (C.I. 0.15, 0.45) | 0.0002 |
| % Monocytes (n=149) | 0.09 (C.I. -0.08, 0.25) | 0.29 |
| % Eosinophils (n=149) | 0.15 (C.I. -0.01, 0.31) | 0.07 |
| % Basophils (n=149) | 0.02 (C.I. -0.15, 0.18) | 0.83 |
| C-reactive protein (n=211) | -0.39 (C.I. -0.51, -0.27) | <0.0001 |
| Erythrocyte sedimentation rate (n=116) | -0.31 (C.I. -0.47, -0.13) | 0.0008 |
| Total proteins (n=137) | 0.16 (C.I. -0.01, 0.32) | 0.06 |
| Albumin (n=88) | 0.47 (C.I. 0.28, 0.62) | <0.0001 |

**Supplementary Table 3.** Correlation analysis between cFAP concentration and routine inflammatory markers from blood analyses in patients with IBD

| **Variable** | **CD (n=21)** |
| --- | --- |
| **Age (mean ± SD, years)** | 41.5 ± 11.8 |
| **Gender, n (%)** |  |
| Female | 9 (42.8) |
| Male | 12 (57.1) |
| **Smoke** |  |
| Yes | 6 (28.6) |
| No | 15 (71.4) |
| **Family history of CD, n (%)** |  |
| Yes | 3 (14.3) |
| No | 18 (85.7) |
| **Disease duration, n (%)** |  |
| <=10 years | 9 (47.4) |
| > 10 years | 10 (52.6) |
| **Age at diagnosis, n (%)** |  |
| A2 | 15 (71.4) |
| A3 | 5 (23.8) |
| **Montreal location, n (%)** |  |
| L1 | 14 (66.7) |
| L2 | 2 (9.5) |
| L3 | 3 (14.3) |
| L4 | 2 (9.5) |
| **Montreal behavior, n (%)** |  |
| B1 | 1 (4.8) |
| B2 | 12 (57.1) |
| B3 | 8 (38.1) |
| **Perianal disease, n (%)** |  |
| Yes | 4 (20.0) |
| No | 16 (80.0) |
| **Post-operative biological therapy, n (%)** |  |
| Yes | 9 (42.8) |
| No | 12 (57.1) |

**Supplementary Table 4.** Baseline demographic and clinical variables of patients with CD who concluded a regular follow up of at least 12 months post-surgery (n=21) Age of patients is expressed as continuous variable (mean ± SD); all the other variables are shown as total number of subjects and frequency distribution

## Supplementary Figures


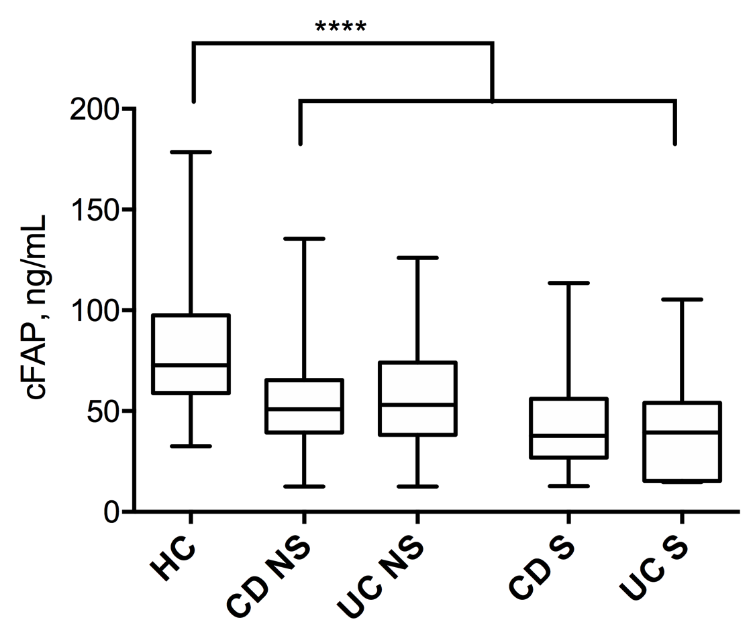


**Supplementary Figure 1.** Box plot displaying the concentration of cFAP in patients with CD and patients with UC attending routine outpatient consultation (CD NS, n=86; UC NS, n=66) or undergoing surgery (CD S, n=96; UC S, n=24) as compared with healthy controls (HC, n=160). Statistical analysis was performed by Student t test, ****p<0.0001


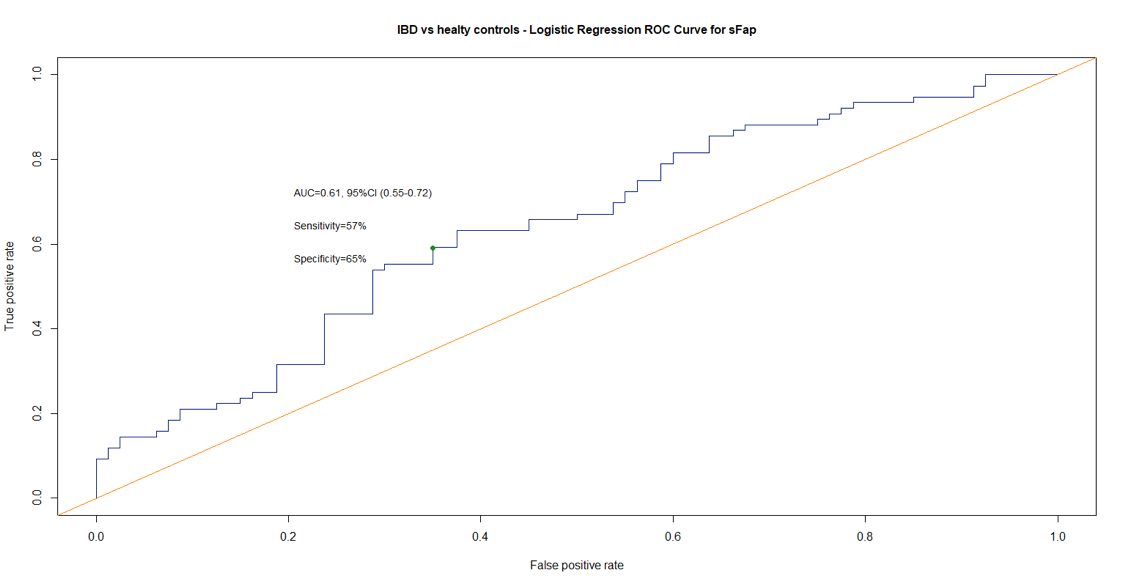


**Supplementary Figure 2.** Receiver operating characteristic (ROC) curve for cFAP level’s accuracy computed on the validation cohort (50% of the original dataset)
